# Feasibility and safety of a 6-month outpatient rehabilitation program combining therapeutic electrical stimulation using belt electrodes and nutritional management in patients with rheumatoid arthritis: a pilot study

**DOI:** 10.3389/fresc.2026.1920901

**Published:** 2026-10-02

**Authors:** Norikazu Hishikawa, Shogo Toyama, Koshiro Sawada, Suzuyo Ohashi, Yasuo Mikami

**Affiliations:** 1 Department of Rehabilitation Medicine, Graduate School of Medical Science, Kyoto Prefectural University of Medicine, Kyoto, Japan; 2 Department of Orthopaedics, Graduate School of Medical Science, Kyoto Prefectural University of Medicine, Kyoto, Japan; 3 Meiji University of Integrative Medicine, Kyoto, Japan

**Keywords:** lower limb, nutritional management, outpatient rehabilitation program, rheumatoid arthritis, sarcopenia, therapeutic electrical stimulation

## Abstract

**Background:**

Although resistance training with nutritional intervention is recommended for sarcopenia, effective programs are often difficult to implement in patients with rheumatoid arthritis (RA). This pilot study aimed to assess the feasibility and safety of a 6-month outpatient rehabilitation program combining therapeutic electrical stimulation (TES) using belt electrodes with nutritional management in patients with RA.

**Methods:**

This pilot study was conducted as a prospective observational study with a single-arm before–after framework and enrolled outpatients with RA in remission or with low disease activity. Participants received an outpatient rehabilitation program as part of routine clinical practice once or twice weekly over 6 months, including TES applied to the lower limbs using belt electrodes (20 min per session; 20 Hz; maximum tolerable intensity) and the daily intake of an L-leucine-enriched protein-containing nutritional supplement. Feasibility and safety were evaluated based on study completion, TES session frequency, verbally confirmed adherence to nutritional supplementation, adverse events documented during the program, and maintenance of disease activity. Lower-limb lean mass, knee extensor muscle strength, and gait speed were assessed as exploratory clinical outcomes at baseline and after 6 months.

**Results:**

Of the 14 recruited outpatients with RA, 10 completed both the 6-month program and the final outcome assessment, corresponding to a study completion rate of 71.4%. Participants received a median of 34.5 TES sessions over 6 months. As exploratory clinical outcomes, the median paired changes in lower-limb lean mass, knee extensor muscle strength, and gait speed were 0.08 kg (95% confidence interval [CI], 0.00 to 0.25; *P* = 0.043), 1.58 kgf (95% CI, −0.35 to 7.25; *P* = 0.084), and 0.18 m/s (95% CI, −0.04 to 0.24; *P* = 0.084), respectively. Disease activity remained within the low disease activity to remission range and did not significantly change during the 6-month observation period (*P* = 0.214). No joint-related or cardiovascular adverse events were documented.

**Conclusions:**

This pilot study provides preliminary information on the feasibility and safety of a 6-month outpatient rehabilitation program combining TES using belt electrodes and nutritional management in patients with RA. These findings may inform the design of future studies.

## 1 Introduction

Advances in pharmacological therapies have substantially improved the management of rheumatoid arthritis (RA) (1). However, many patients with RA have sarcopenia, characterized by the loss of skeletal muscle mass and decreased muscle strength (2, 3), primarily attributed to aging, chronic inflammation, repeated exposure to glucocorticoids, and physical inactivity (2–4). Patients with RA and sarcopenia often have accelerated joint destruction, limitations in activities of daily living (ADL), and a higher incidence of falls resulting in fractures (3–5). In addition to pharmacological therapies, non-pharmacological treatments, such as rehabilitation treatment, are often administered simultaneously to manage sarcopenia. Adequate pharmacological therapy remains the cornerstone of RA management, and non-pharmacological interventions such as rehabilitation treatment are intended to complement optimized drug treatment (6). Although advances in pharmacological therapies, including molecular targeted therapies [e.g., biologic and targeted synthetic disease-modifying antirheumatic drugs (DMARDs)], have enabled glucocorticoid dose reduction in many patients with RA, they do not diminish the importance of rehabilitation treatment for maintaining skeletal muscle mass and muscle strength.

Rehabilitation treatment is recommended for improving physical function in patients with RA in the treatment guidelines of multiple countries (6–8). However, these recommendations are largely based on evidence derived from high-intensity and high-frequency exercise interventions, including resistance and aerobic training. Although these exercise interventions are generally considered safe and effective for patients with RA, some individuals may experience difficulties participating in high-intensity training programs because of joint pain, advanced joint damage, or fear of symptom exacerbation (9). In addition, the exercise frequency recommended in current guidelines (e.g., three sessions per week), together with the time required to travel to rehabilitation facilities, is often impractical in real-world clinical practice. In Japan, systemic constraints in clinical practice frequently result in an insufficient dose of rehabilitation treatment for outpatients with RA (e.g., typically only one or two sessions per week). Hence, standard sarcopenia management in RA—typically consisting of a combination of resistance training and nutritional interventions, including an adequate intake of protein and essential amino acids (10)—is often difficult to implement in routine outpatient practice. As a result, maintaining skeletal muscle mass and muscle strength remains a major clinical challenge in patients with RA. Therefore, alternative rehabilitation strategies aimed at maintaining or improving skeletal muscle mass and muscle strength with a lower burden are needed for this population.

Therapeutic electrical stimulation (TES) induces involuntary muscle contractions and may serve as an alternative muscle-strengthening technique when exercise capacity is restricted or limited (11). Previous studies have applied TES, including neuromuscular electrical stimulation, in patients with RA; however, the available evidence remains limited, and the stimulation area in conventional approaches is typically confined to localized muscle groups (12, 13). A novel TES system that uses belt electrodes provides a wider stimulation area over the lower limbs than conventional electrodes and reduces skin discomfort, enabling the simultaneous activation of multiple muscle groups (14, 15). Animal studies using collagen-induced arthritis models have demonstrated that TES using belt electrodes prevents muscle atrophy without exacerbating arthritis, supporting further clinical evaluation of this approach in RA (16). Therefore, when combined with nutritional management, TES using belt electrodes may serve as a practical alternative to resistance training in outpatient settings for patients with RA. However, there is a paucity of evidence regarding the feasibility and safety of TES using belt electrodes combined with nutritional management in human patients with RA, highlighting the need for translational clinical studies.

This pilot study aimed to assess the feasibility and safety of a 6-month outpatient rehabilitation program combining TES using belt electrodes and nutritional management in patients with RA with relatively well-controlled disease activity. Exploratory changes in lower-limb lean mass, knee extensor muscle strength, and gait speed were also examined to inform the design of future larger-scale studies.

## 2 Materials and methods

### 2.1 Study design and ethics

This pilot study was conducted as a prospective observational study with a single-arm before–after framework. Although outcomes following the rehabilitation program were prospectively evaluated, participation in the program was determined as part of routine clinical care based on the treating rheumatologist's clinical judgment and not by the research protocol. Therefore, the study was classified as observational rather than interventional. The study protocol was approved by the Ethics Review Board of Kyoto Prefectural University of Medicine (ERB-C-2547-5) and was prospectively registered with the University Hospital Medical Information Network Clinical Trials Registry (UMIN-CTR: UMIN000049735).

The study was conducted in accordance with the Declaration of Helsinki. The requirement for written informed consent was waived by the Ethics Review Board because this study was conducted within routine clinical practice. The rehabilitation program was not introduced specifically for research purposes. Therefore, an opt-out consent procedure was implemented in accordance with relevant ethical guidelines and national regulations. In addition, all participants received an oral explanation of the study, and their agreement to participate was documented in the medical records. This study was reported in accordance with the Strengthening the Reporting of Observational Studies in Epidemiology (STROBE) statement, where applicable (17).

### 2.2 Participants

Patients with RA who were receiving outpatient rehabilitation between December 2022 and January 2024 were assessed for inclusion in this study. RA was diagnosed according to the 2010 classification criteria of the American College of Rheumatology and the European League Against Rheumatism (18). The inclusion criteria included age ≥ 20 years, disease activity classified as remission (scores ≤ 3.3) or low disease activity (scores > 3.3 to ≤ 11), according to the Simplified Disease Activity Index (SDAI) (19), and receipt of outpatient rehabilitation once or twice weekly for at least 6 months, with the frequency determined according to routine clinical practice. The exclusion criteria included pregnancy or implantation of a cardiac pacemaker; patients with an increase in prednisolone dose of ≥ 5 mg; the requirement for intra-articular steroid injections into a weight-bearing joint, as determined by the attending physician; a history of central nervous system, cardiopulmonary, metabolic, or psychiatric disorders; and patients within 6 months of RA onset and those deemed inappropriate for participation by the attending physician. Previous initiation of biologic agents or Janus kinase inhibitors was not an exclusion criterion; however, participants who initiated or switched biologic or targeted synthetic DMARDs during the observation period were to be excluded from the analysis because such treatment changes could confound the assessment of outcomes. Radiographic severity, including the degree of joint destruction (e.g., Steinbrocker's stage), was not used as an inclusion or exclusion criterion, because the intervention was designed to induce muscle contractions with minimal joint movement and joint loading and was, therefore, considered suitable for patients across a broad spectrum of disease severity.

### 2.3 Data collection

The demographic and clinical data collected included age, sex, body mass index, body fat percentage, disease duration, drug regimen, disease activity, serology profile (rheumatoid factor), joint destruction (Steinbrocker's stage), nutritional status, ADL, lower-limb lean mass, lower-limb muscle strength, and gait speed. The disease activity, nutritional status, and ADL were assessed using the SDAI, the Geriatric Nutritional Risk Index (20), and the Health Assessment Questionnaire-Disability Index (21), respectively. The Geriatric Nutritional Risk Index values were classified into the following categories: no nutritional risk (≥ 98), low risk (92–97), moderate risk (82–91), and major risk (< 82). The Health Assessment Questionnaire-Disability Index scores range from 0 to 3, with higher scores indicating more serious disability.

Body fat percentage and segmental lean mass of the lower limbs were assessed using a multifrequency bioelectrical impedance analyzer (InBody S10, InBody Japan Inc., Tokyo, Japan). Body composition measurements were performed under standardized conditions, with reference to recommended procedures for bioelectrical impedance analysis (22). Lower-limb muscle strength was assessed using knee extensor muscle strength as an indicator. Participants performed maximal isometric knee extension for approximately 5 s in a seated position, and the force was measured using a hand-held dynamometer (*μ*Tas F-1, Anima Co., Ltd., Tokyo, Japan). For both lower-limb lean mass and knee extensor muscle strength, the higher value obtained from the right or left side was used for analysis to minimize the potential influence of unilateral joint involvement or disuse. Gait speed was assessed using the 6-Meter Walk Test. The time required to complete the test was measured using a stopwatch, and gait speed was calculated by dividing the walking distance by the recorded time.

### 2.4 Outpatient rehabilitation program

An outpatient rehabilitation program, including TES using belt electrodes and nutritional management, was provided as part of routine clinical practice according to previously reported procedures (23). Specifically, participants received therapist-supervised outpatient rehabilitation sessions as part of routine clinical practice for 40 min once or twice weekly over a 6-month period. Because the frequency of outpatient rehabilitation was determined according to routine clinical practice and individual participant circumstances, a fixed number of TES sessions was not prespecified for each participant. The rehabilitation program comprised lower-limb stretching exercises, gait training, and TES using belt electrodes. Stretching exercises included lower-limb range-of-motion and flexibility exercises targeting the hip, knee, and ankle joints. Gait training consisted of supervised walking practice tailored to each participant's physical condition. TES was administered for 20 min per session using a commercially available electrical stimulator (G-TES®; Homer Ion Laboratory Co., Ltd., Tokyo, Japan). Figure 1 shows the placement of the belt electrodes used in this study. Five elastic belt electrodes were placed around the waist, above the knees, and above the ankles to enable the simultaneous stimulation of multiple lower limb muscle groups. Electrical stimulation was delivered in disuse mode (frequency: 20 Hz; pulse width: 250 μs; duty cycle: 5 s on and 2 s off) to facilitate muscle strengthening. The stimulation intensity was set at the maximum level tolerated by each participant, and was reassessed and adjusted as needed every five minutes during each session to ensure that it remained at the maximum tolerable level.

**Figure 1:**
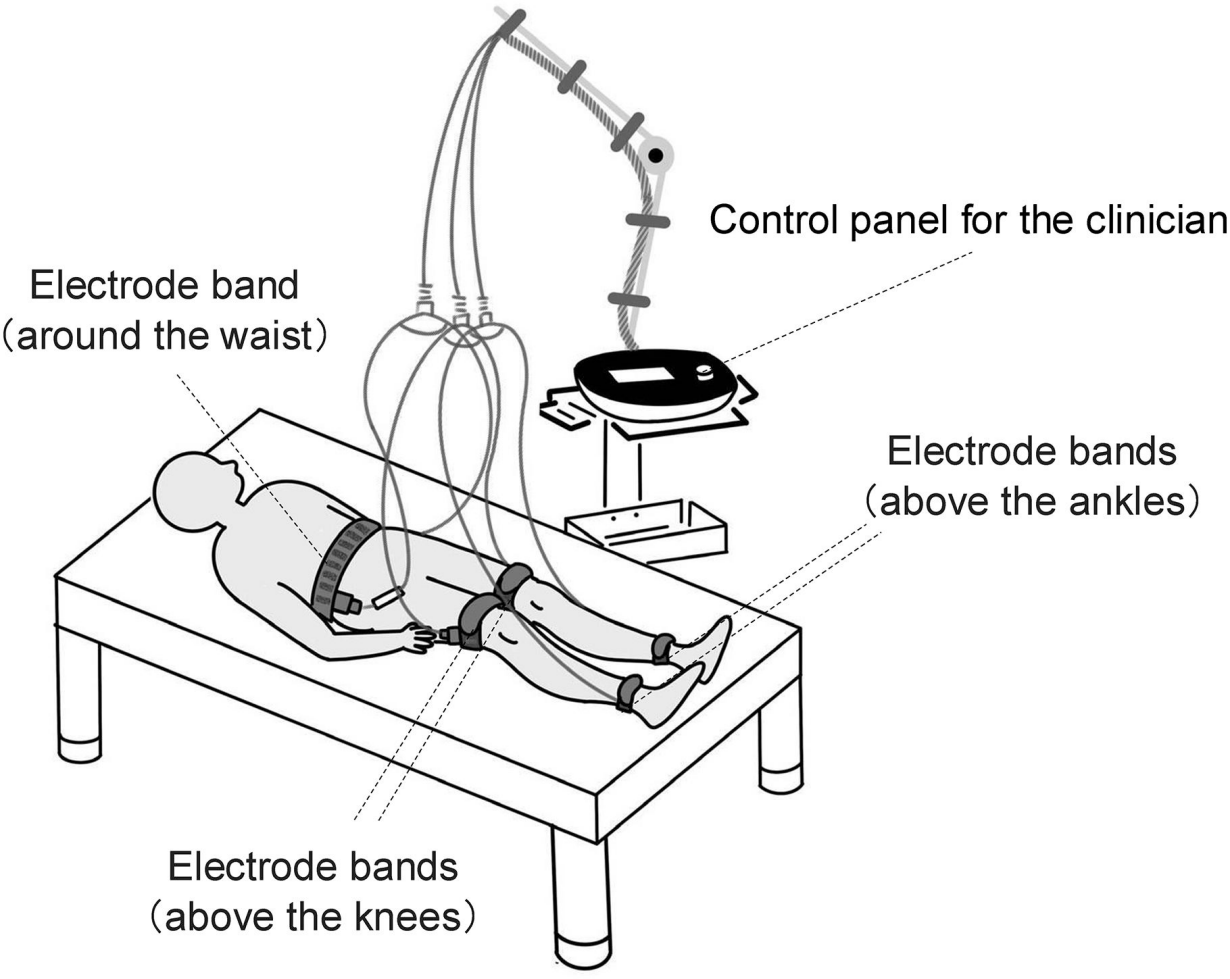
Configuration of the TES system using belt electrodes for the lower limbs. Five belt electrodes were placed around the waist and lower limbs (above both knees and above both ankles) to allow simultaneous bilateral lower limb stimulation. Reproduced from “Configuration of the TES system using belt electrodes” by Norikazu Hishikawa, Shogo Toyama, Koshiro Sawada, Motonori Kubo, Suzuyo Ohashi and Yasuo Mikami, licensed under CC BY 4.0.

Each participant also received nutritional management. Participants were instructed to consume one serving of a leucine-enriched nutritional supplement (HINEX® REHADAYS; Otsuka Pharmaceutical Factory, Inc., Tokushima, Japan) daily after breakfast for 6 months. Each 125 mL serving contained 160 kcal of energy, 11.0 g of protein, and 2300 mg of L-leucine. At each routine outpatient rehabilitation visit, the treating therapist verbally confirmed participants’ adherence to the nutritional supplementation, and nutritional supplements sufficient to last until the next scheduled visit were provided.

Feasibility and safety outcomes included study completion, TES session frequency, adherence to nutritional supplementation, adverse events documented during the program, and maintenance of disease activity, as assessed using the SDAI. Study completion was defined as completion of both the 6-month program and the final outcome assessment. Adverse events, including clinically apparent symptoms potentially related to the intervention (e.g., exacerbation of joint pain, skin irritation, muscle soreness, and fatigue), were monitored before and after outpatient rehabilitation sessions through participant questioning and clinical observation and were recorded in the medical records when identified. Adherence to nutritional supplementation was assessed by verbal confirmation from participants at each routine outpatient rehabilitation visit; quantitative adherence data were not systematically recorded.

### 2.5 Statistical analysis

Demographic and clinical data are presented as medians with lower and upper quartiles or numbers with percentages. Where applicable, baseline demographic and clinical characteristics were also descriptively summarized according to study completion status, without formal between-group statistical testing. The Wilcoxon signed-rank test was used to compare outcomes within participants before and after 6 months of the outpatient rehabilitation program. For these outcomes, paired changes were calculated as the 6-month value minus the baseline value, and the median paired changes and their 95% confidence intervals (CIs) were estimated using bias-corrected and accelerated bootstrap resampling with 100,000 resamples. The statistical significance level was set to 5%. Effect sizes were classified as small, medium, or large, using *r* values of 0.1, 0.3, and 0.5 as the respective thresholds (24). Statistical analyses were performed using IBM SPSS Statistics version 29.0 (IBM Corp., Armonk, NY, USA) and R version 4.6.1 (R Foundation for Statistical Computing, Vienna, Austria). As this was a pilot study primarily intended to evaluate feasibility and safety and to generate preliminary estimates for future studies, no formal sample size calculation was performed.

## 3 Results

Fourteen outpatients with RA were recruited, of whom 10 completed both the 6-month program and the final outcome assessment, corresponding to an observed study completion rate of 71.4%. Of the remaining four participants, two had difficulty maintaining regular outpatient attendance because of hot weather, one discontinued the program, including TES, after receiving a new diagnosis of cancer at another hospital, and one completed the 6-month program but had no available 6-month outcome data. The 10 participants with complete baseline and 6-month outcome data were included in the before–after analysis of exploratory clinical outcomes (Figure 2). Baseline demographic and clinical characteristics of all 14 enrolled participants, stratified by completion status, are presented in Table 1. Given the small number of non-completers, these characteristics are presented descriptively. Among the 10 completers, there were four men (40%) and six women (60%). Most completers were older adults with relatively short disease duration and low disease activity. They exhibited mild ADL limitations but had no apparent nutritional risk. Radiographic severity varied among the completers, and patients with different degrees of joint destruction were included. All completers were receiving conventional synthetic, biologic, or targeted synthetic DMARDs. Three completers (30%) used methotrexate, and four (40%) used low-dose oral prednisolone at doses ≤7 mg/day.

**Figure 2:**
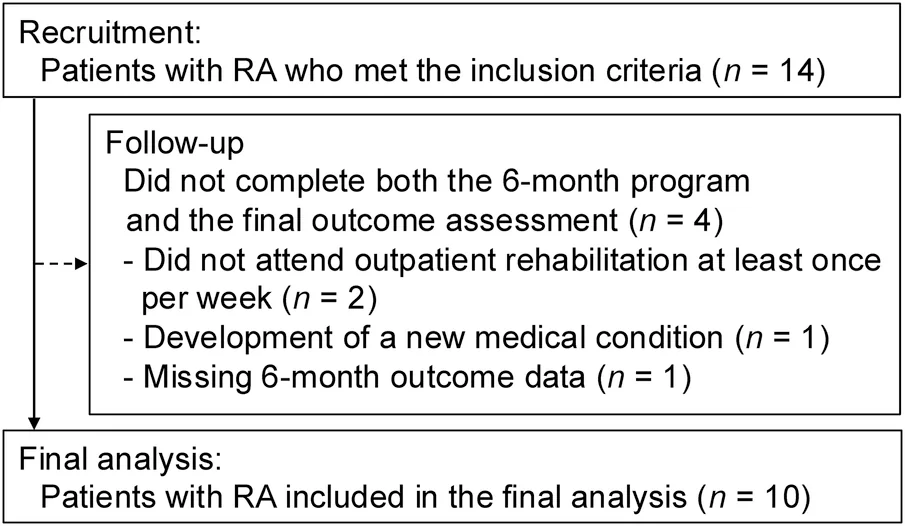
Participant flow diagram showing recruitment, non-completion, and final analysis.

**Table 1:** Baseline demographic and clinical characteristics of enrolled participants according to study completion status.

Characteristic	All participants (*n* = 14)	Study completers (*n* = 10)	Study non-completers (*n* = 4)
Age, years	73.5 (71.0, 79.8)	73.5 (71.3, 75.8)	71.5 (58.3, 81.8)
Sex, male/female, *n* (%)	4/10 [29/71]	4/6 [40/60]	0/4 [0/100]
Body mass index, kg/m^2^	22.1 (20.1, 24.9)	23.8 (20.4, 25.5)	20.2 (19.5, 21.3)
Body fat, %	28.9 (25.3, 33.2)	28.9 (26.5, 34.8)	26.1 (21.3, 30.6)
Disease duration, years	3.5 (2.0, 5.0)	2.5 (2.0, 3.8)	5.0 (4.8, 6.3)
Disease activity			
SDAI, score	4.9 (3.0, 7.2)	5.2 (4.8, 7.2)	1.9 (1.0, 4.5)
Serology profile			
Rheumatoid factor, IU/mL	13.5 (6.0, 52.3)	21.0 (8.0, 76.8)	6.0 (3.8, 8.5)
Joint destruction			
Steinbrocker's stage,	3/7/0/4	3/5/0/2	0/2/0/2
I/II/III/IV, *n* (%)	[21/50/0/29]	[30/50/0/20]	[0/50/0/50]
Nutritional status			
GNRI	102.6 (98.5, 109.5)	104.7 (101.0, 109.5)	97.8 (96.9, 101.1)
ADL			
HAQ-DI	0.4 (0.1, 0.5)	0.4 (0.1, 0.5)	0.4 (0.2, 0.8)

Data are presented as medians (lower quartile, upper quartile) or numbers [percentages]. SDAI, Simplified Disease Activity Index; GNRI, Geriatric Nutritional Risk Index; ADL, activities of daily living; HAQ-DI, Health Assessment Questionnaire-Disability Index.

Participants received a median of 34.5 (29.4, 41.0) TES sessions over 6 months, corresponding to approximately 1.3 sessions per week. Figure 3 shows the within-participant changes in outcome measures before and after the 6-month outpatient rehabilitation program. Lower-limb lean mass was 5.4 (4.9, 7.9) kg at baseline and 5.6 (4.9, 8.2) kg at 6 months. The median paired change was 0.08 kg (95% CI, 0.00 to 0.25; *P* = 0.043; *r* = 0.640). Knee extensor muscle strength changed from 31.5 (19.1, 43.3) kgf to 36.6 (19.2, 49.0) kgf, with a median paired change of 1.58 kgf (95% CI, −0.35 to 7.25; *P* = 0.084; *r* = 0.546). Gait speed changed from 1.0 (0.7, 1.0) m/s to 1.0 (0.7, 1.2) m/s, with a median paired change of 0.18 m/s (95% CI, −0.04 to 0.24; *P* = 0.084; *r* = 0.546). The SDAI remained within the low disease activity to remission range, decreasing from 5.2 (4.8, 7.2) at baseline to 2.9 (2.4, 6.7) at 6 months, without a statistically significant change during the 6-month observation period (*P* = 0.214, *r* = 0.393). No joint-related or cardiovascular adverse events were documented during the 6-month outpatient rehabilitation program.

**Figure 3:**
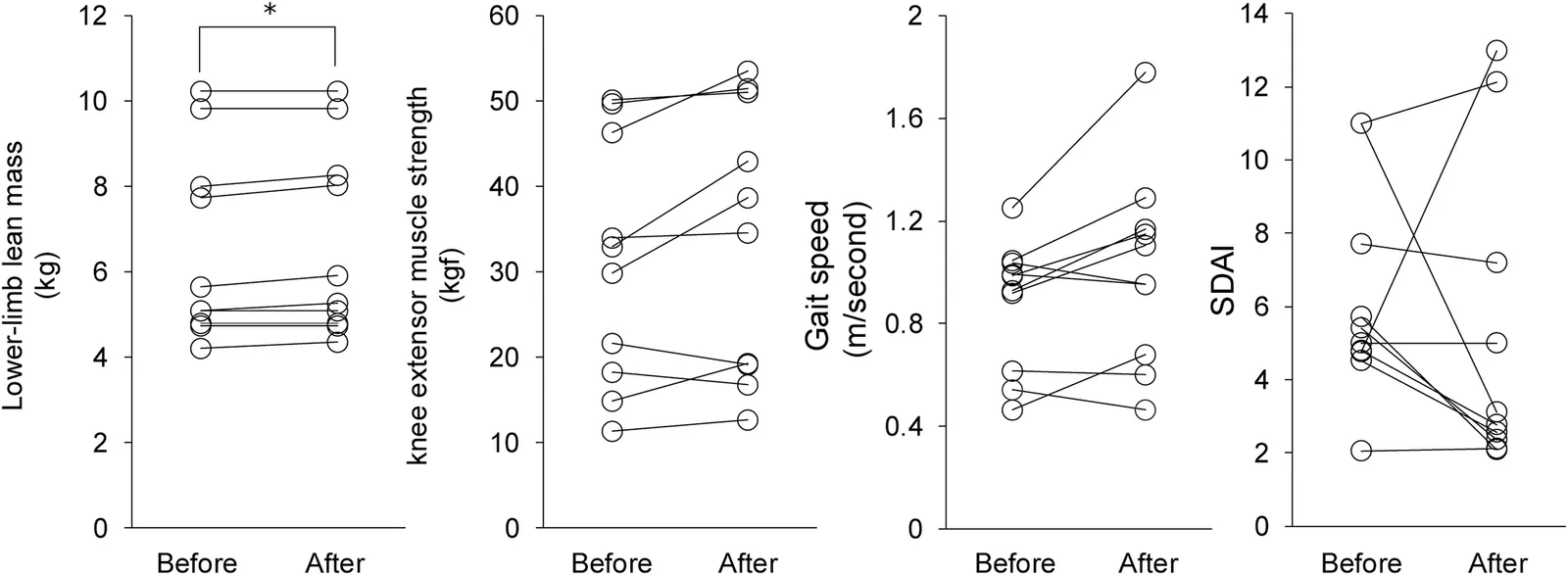
Outcome measures before and after the 6-month outpatient rehabilitation program.

## 4 Discussion

The present pilot study evaluated the feasibility and safety of a 6-month outpatient rehabilitation program combining TES using belt electrodes with nutritional management in patients with RA. Ten of the 14 enrolled participants completed both the 6-month program and the final outcome assessment. Disease activity did not worsen, and no joint-related or cardiovascular adverse events were documented among these participants. These findings provide descriptive information on the implementation of this multicomponent program in a real-world outpatient setting. Exploratory changes were also observed in lower-limb lean mass, knee extensor muscle strength, and gait speed over the 6-month observation period; however, these findings should be interpreted as hypothesis-generating rather than as evidence of efficacy.

The importance of exercise therapy for patients with RA is clear (6–8); however, its implementation can be challenging, particularly for individuals with joint pain, advanced joint damage, or fear of symptom exacerbation (9). For exercise therapy to be widely implemented as a core component of non-pharmacological management in RA, its feasibility from the patient's perspective warrants consideration. Adherence may depend on whether the prescribed regimen can be followed without imposing an excessive burden on the patient. Furthermore, increasing skeletal muscle mass generally requires sufficient muscle contraction and mechanical loading to stimulate anabolic processes in skeletal muscle (25). Consequently, there is a clinical need to explore alternative methods of inducing muscle contractions that require minimal effort.

The present study evaluated a 6-month multicomponent rehabilitation program under routine outpatient clinical conditions. As described above, outpatient rehabilitation for patients with RA is often constrained by systemic and logistical limitations that restrict the frequency and intensity of therapeutic interventions. Within this context, participants included in the final analysis received a median of 34.5 TES sessions during the 6-month study period. Together with the observed study completion rate, this level of intervention exposure provides descriptive information on the implementation of the program in routine outpatient practice.

In addition to feasibility, disease activity assessed using the SDAI as part of risk management remained within the low disease activity to remission range at baseline and at 6 months. Furthermore, no joint-related or cardiovascular adverse events were documented during the 6-month observation period. Although these observations are relevant to the safety assessment, their interpretation is limited by the small sample size and the limitations of adverse-event assessment. Because TES using belt electrodes can induce muscle contractions with minimal joint movement or external mechanical loading, its potential applicability in patients with RA who have joint pain or concerns regarding mechanical stress warrants further investigation.

Exploratory clinical outcomes were also assessed. Lower-limb lean mass changed from a median of 5.4 kg at baseline to 5.6 kg at 6 months, with a median paired change of 0.08 kg. The magnitude of this median paired change was small, and further studies are needed to determine its clinical relevance. Similarly, changes in knee extensor muscle strength and gait speed were observed over the 6-month period. Given the small sample size, the absence of a control group, and the assessment of multiple exploratory clinical outcomes, these findings should be interpreted as exploratory and may inform the design, outcome selection, and planning of future controlled studies.

Because the present program combined TES with nutritional management, the potential contribution of nutritional support should also be considered. Nutritional management, including leucine-enriched protein supplementation, was incorporated as an adjunct to TES to support skeletal muscle anabolic responses. Adequate protein intake is recognized as a vital component of maintaining and restoring muscle function during exercise interventions (26). Although the participants in the present study, as a group, did not exhibit overt nutritional risks, patients with RA are generally prone to malnutrition, the progression of which is attributed to excessive protein catabolism induced by inflammatory cytokines and skeletal muscle loss associated with functional impairment (27, 28). In outpatient care for patients with RA, where the frequency of rehabilitation treatment is limited, nutritional support may represent a complementary component of rehabilitation management. Because both exercise-based interventions and nutritional support are recommended components of sarcopenia management (10), the present study evaluated the feasibility of a multicomponent rehabilitation program rather than the independent effects of its individual components. Further studies with comprehensive dietary assessments are needed to clarify the contribution of nutritional management within this multicomponent program.

Several limitations of this study should be acknowledged. First, no *a priori* feasibility threshold or progression criterion was prespecified. Therefore, the observed completion rate, TES session frequency, and other feasibility-related findings should be interpreted descriptively. Second, the small sample size limits the precision and generalizability of the findings. In addition, the absence of a control group precludes causal inference regarding the observed changes. Because the program consisted of multiple components, including TES, conventional rehabilitation, and nutritional supplementation, the contribution of each component to the observed outcomes cannot be determined. Furthermore, natural variability, changes in habitual physical activity, and regression to the mean cannot be excluded as potential contributors to the observed changes. Accordingly, the exploratory clinical outcomes should not be interpreted as evidence of efficacy. Future controlled studies with appropriate comparator groups are required to evaluate the efficacy of the program and to determine the added value of TES beyond conventional rehabilitation and nutritional management. Third, although body composition measurements were performed under standardized conditions, bioelectrical impedance analysis can be influenced by factors such as hydration status, food intake, recent physical activity, and other premeasurement conditions (22). In addition, the use of the higher value from either leg for lower-limb lean mass, although intended to minimize the potential influence of unilateral joint involvement or disuse, may limit comparability with studies using other approaches to derive bilateral lower-limb lean mass. Therefore, given the small absolute change observed in lower-limb lean mass, the potential influence of measurement variability should be considered when interpreting this finding. Fourth, although TES session frequency was recorded, a fixed number of scheduled TES sessions was not prespecified for each participant; therefore, adherence to TES could not be quantified as the proportion of scheduled sessions completed. In addition, adherence to nutritional supplementation was assessed by verbal confirmation during routine outpatient rehabilitation visits, and quantitative adherence data, such as the number or proportion of supplements actually consumed, were not systematically recorded. Future studies should incorporate more rigorous and objective measures of nutritional adherence, such as intake logs or counts of returned unused supplements. Adverse events were not prospectively defined or graded according to a standardized classification system. Although participants were asked about symptoms before and after rehabilitation sessions, a standardized adverse-event checklist or severity grading system was not used. Accordingly, the absence of documented adverse events should be interpreted in light of these limitations. In addition, patient-reported outcomes, including pain intensity, patient satisfaction, and TES tolerability, were not systematically collected. These measures should be incorporated into future studies to provide a more comprehensive assessment of feasibility, acceptability, and tolerability. Fifth, most participants had low disease activity and relatively mild disability, which may limit the generalizability of the findings to patients with more active RA or greater functional impairment. Furthermore, participants were not selected on the basis of a formal diagnosis of sarcopenia according to established diagnostic criteria; therefore, the findings should not be generalized specifically to patients with RA-associated sarcopenia. Finally, further studies with longer follow-up periods are needed to evaluate the longer-term feasibility and clinical outcomes of the program. Despite these limitations, the present findings may inform the design of future controlled studies with predefined feasibility criteria, more rigorous adherence and safety assessments, and appropriate comparator groups.

## 5 Conclusion

This pilot study provides preliminary information on the feasibility and safety of a 6-month outpatient rehabilitation program combining TES using belt electrodes with nutritional management in patients with RA. No joint-related or cardiovascular adverse events were documented in this small cohort during the 6-month program. These findings provide a basis for further investigation of this multicomponent program in controlled studies with more rigorous adherence and safety assessments.

## Data Availability

The raw data supporting the conclusions of this article will be made available by the authors, without undue reservation.
